# Philadelphia County Dental Society

**Published:** 1893-02

**Authors:** W. A. Phreaner

**Affiliations:** Secretary; 1415 Walnut Street; Philadelphia


### PHILADELPHIA COUNTY DENTAL SOCIETY.

PHILADELPHIA, December 14, 1892.

TO THE EDITOR OF THE INTERNATIONAL DENTAL JOURNAL:

SIR,—In the INTERNATIONAL DENTAL JOURNAL for January, 1892, appeared an editorial entitled "A Legal Outrage." This editorial is what it styles the acts of others,—"an open violation of all professional practice" and ethics. It is filled with statements which are unjust and untrue. When the Committee on Enforcement of Law of the Pennsylvania State Dental Society, or the Legal Committee of the Philadelphia County Dental Society, upon whom have been placed the solemn obligations to enforce the laws of the Commonwealth of Pennsylvania, proceeded to the discharge of their duty, their action can hardly be stigmatized as "officious intermeddling of individuals and societies," or as "tyrannical proceedings." An examination of the charter of the Philadelphia County Dental Society will show that it is not "modelled after law and order associations," and that its "sole object" is not "the prosecution of violators of the dental law of Pennsylvania." The Philadelphia County Dental Society has not for one moment, or in a single instance, "overstepped the bounds laid out for its government." These and other inaccuracies are embodied in the editorial in question, and it concludes with the serious charge that the gentlemen of these committees have, "under the guise of a public duty, openly violated all professional practice," and makes a call upon societies "to promptly place such individuals beyond the pale of professional recognition." The Philadelphia County Dental Society has waited in the hope that your own sense of right would lead you to rectify these misstatements, and they especially trusted that this would be done after the attention of the Pennsylvania State Dental Society was called to them this summer. The *amende honorable* has not been made, and at a special meeting of the Board of Directors of the Philadelphia County Dental Society, held on December 13, the Secretary was instructed to address this letter to you, and request that you send by return mail the assurance that a suitable apology will be inserted in the next issue of the JOURNAL.

Sincerely yours,
W. A. PHREANER,
*Secretary.*

1415 WALNUT STREET.

[REMARKS.—We give the "Philadelphia County Dental Society" the benefit of the publication of the above peculiar communication.

We have not heard from the Pennsylvania State Dental Society, but we can assure our correspondent that when that body furnishes this journal with its criticism, it will be received with the respect due such an organization.

Inasmuch as the Philadelphia County Dental Society was not named in the editorial alluded to, and as our views have not undergone any modification by the *lapse of a year*, we see no necessity for a compliance with the demand for an apology.—ED.]

